# Potential Antiviral Agents from Marine Fungi: An Overview

**DOI:** 10.3390/md13074520

**Published:** 2015-07-22

**Authors:** Soheil Zorofchian Moghadamtousi, Sonia Nikzad, Habsah Abdul Kadir, Sazaly Abubakar, Keivan Zandi

**Affiliations:** 1 Biochemistry Program, Institute of Biological Sciences, Faculty of Science, University of Malaya, 50603 Kuala Lumpur, Malaysia; E-Mails: soheil.zorofchian@gmail.com (S.Z.M.); sonia.nikzad@gmail.com (S.N.); habsah@um.edu.my (H.A.K.); 2 Department of Medical Microbiology, Tropical Infectious Disease Research and Education Center (TIDREC), Faculty of Medicine, University of Malaya, 50603 Kuala Lumpur, Malaysia; E-Mail: sazaly@um.edu.my; 3 The Persian Gulf Marine Biotechnology Research Center, Bushehr University of Medical Sciences, Bushehr 75169, Iran

**Keywords:** natural products, marine fungi, antiviral, review

## Abstract

Biodiversity of the marine world is only partially subjected to detailed scientific scrutiny in comparison to terrestrial life. Life in the marine world depends heavily on marine fungi scavenging the oceans of lifeless plants and animals and entering them into the nutrient cycle by. Approximately 150 to 200 new compounds, including alkaloids, sesquiterpenes, polyketides, and aromatic compounds, are identified from marine fungi annually. In recent years, numerous investigations demonstrated the tremendous potential of marine fungi as a promising source to develop new antivirals against different important viruses, including herpes simplex viruses, the human immunodeficiency virus, and the influenza virus. Various genera of marine fungi such as *Aspergillus*, *Penicillium*, *Cladosporium*, and *Fusarium* were subjected to compound isolation and antiviral studies, which led to an illustration of the strong antiviral activity of a variety of marine fungi-derived compounds. The present review strives to summarize all available knowledge on active compounds isolated from marine fungi with antiviral activity.

## 1. Introduction

The marine world contains approximately one half of all species [1,2]. The vast expanse of the ocean and its unique environment are responsible for the exceptional chemical and biological diversity of marine organisms, with 300,000 described species and far more still to investigate [3]. The fact that less than 0.01%–0.1% of microbial species from the ocean are known to scientists highlights how effectively marine exploration can open up numerous avenues to marine organisms and their active chemical constituents [4]. Virtually all types of marine organisms, including algae, ascidians, bacteria, corals, fungi, and sponges, have come under scientific scrutiny for their natural products [5,6]. As a result of these studies, the ocean provides agrichemicals, cosmetics, enzymes, nutritional supplements, and pharmaceuticals, with great commercial prospects [7,8,9].

Historically, the pivotal role of fungi in different aspects of human life is very pronounced and this is true even in the marine world [10]. Marine fungi belong to the phyla Ascomycota, Bacidomycota, Chytridiomycota, Deuteromycota, and Zygomycota [11]. Evolution of these heterotrophic eukaryotes to degrade different solid substrates helps them to recycle dead plants (e.g., lignan and cellulose) and animal tissues (e.g., chitin and keratin) into the marine ecosystem through decomposition [10,12]. Investigations on marine fungi primarily commenced because of certain infections in the marine environment. Tolerance of some terrestrial species to the conditions of the marine ecosystem, including salt concentration, has made them potent pathogens in the marine world [13]. For instance, pathogenicity of genus *Aspergilus* and *Fusarium solani* contributed to the mortality of the Caribbean sea-fan and the infections of different marine crustaceans, respectively [14,15]. In addition, blue crabs, lobster eggs, and cultured crabs were reported to be infected by *Lagenidium callinectes* [16].

Despite the pathogenicity of certain marine fungi species, mutualistic interactions are the dominant types of relationship found in marine fungi [12]. The life of marine fungi heavily depends on their symbiotic relationships with other marine organisms such as algae and marine invertebrates [17]. For instance, *Turgidosculum ulvae* can only be grown in the thallus of *Blidingia minima*, a green algae [18]. Moreover, different *Penicillium* and *Aspergillus* species in the marine environment are isolated from sponges [19]. Isolation of these species requires the collection of the supporting material or host marine organism. Therefore, investigations on marine fungi confront the serious impediment of preserving samples until extraction [17].

As scientific interest has been sparked in marine microorganisms, fungi and their metabolites have begun to be recognized for their potent biological activities in the past few decades. Some of these metabolites give marine fungi the superiority to adapt to extreme habitats, compete for substrates, and ward off threats [20]. Moreover, fungi metabolites may be affected by their source of isolation, including sponges or other invertebrates, whose tissues they are harboring on or living in. Compounds isolated from marine fungi elicited promising assorted biological activities, especially anticancer and antidiabetic properties. However, other pharmaceutical activities have also reported, including cell cycle inhibition, kinase and phosphatase inhibition, antioxidant, neuritogenic, anti-inflammatory, antiplasmodial, and antiviral activities [12,21,22,23,24].

## 2. Marine Fungi and Their Antiviral Activity

**Figure 1:**
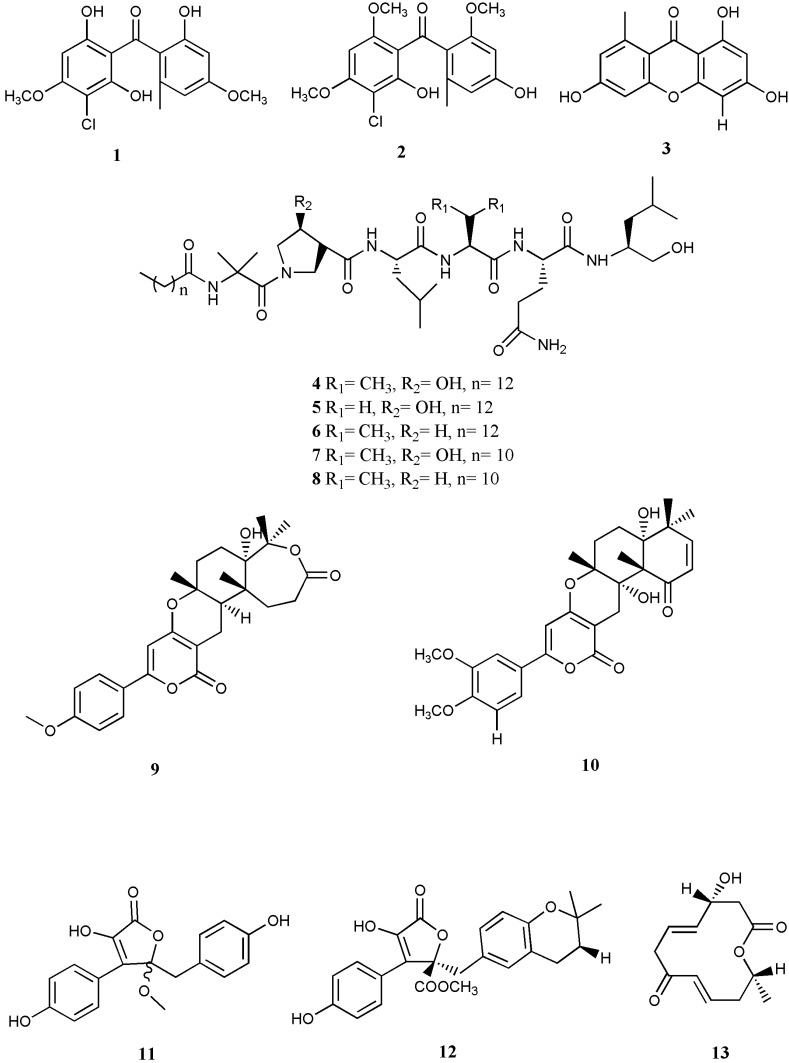

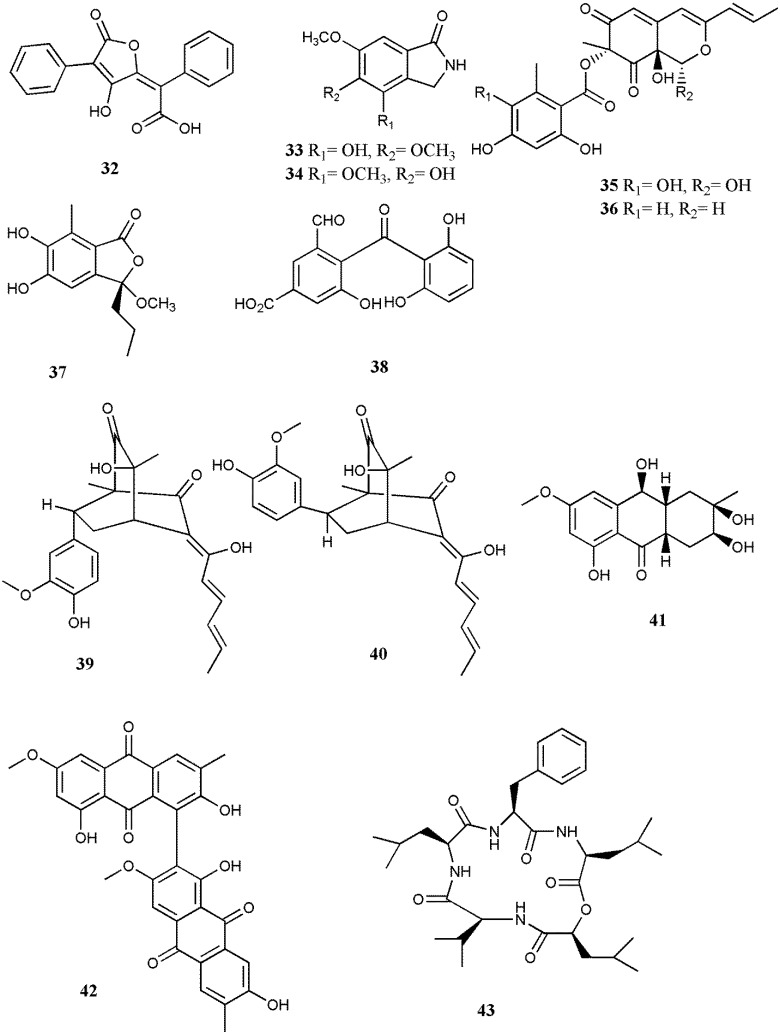

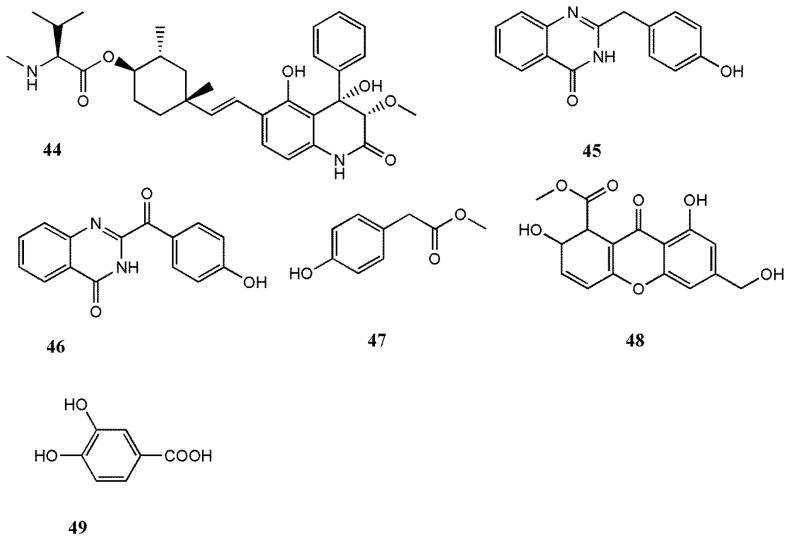
Chemical structures of marine fungi-derived compounds with antiviral activity.

Viruses encompass a prodigious group of microorganisms causing assorted infectious diseases. Recent scientific studies triumphantly reported new antiviral agents, which generally inhibit the virus replication cycle through affecting the important host cell factor(s) for virus replication and/or viral elements [25]. Despite the marked development in antiviral pharmaceuticals over the past few decades, patients suffering from viral infections are severely afflicted with treatment failure mostly because of the emergence of recombinant viruses, drug resistance, and cell toxicity [26,27,28]. Moreover, the widespread occurrence of chronic viral infectious diseases, including human immunodeficiency virus (HIV) and viral hepatitis, clearly warrants exploration of new therapeutic agents with higher efficiency and diminished side effects. From 1981 to 2010, only 18 out of 110 established antiviral drugs were biological derivatives of natural products, including laninamivir, oseltamivir, and zanamivir. Nonetheless, the portion of synthetic drugs made of modified nucleosides and peptidomimetics, *etc.* or mimicking natural products were much higher. This highlights the potential role of natural products in the further establishment of new antiviral agents [29]. The focus on the antiviral potential of compounds isolated from marine fungi came to light in 1998 after the isolation of stachyflin from *Stachybotrys* sp. RF-7260 by Taishi and colleagues; it had promising antiviral activity against influenza A virus (H1N1) [30]. Until 2006, a limited number of compounds with antiviral activity was reported, as reviewed by Bhadury and colleagues [3]. However, over the last decade, numerous compounds with promising antiviral activities against various viruses were isolated from marine fungi. Later in this review, we outline the types of viruses which were subjected to marine fungi-derived compounds and elucidate their source of isolation and antiviral potential (Figure 1, Table 1).

### 2.1. Enterovirus-71 (EV-71)

After the virtual eradication of poliovirus, enterovirus 71 (EV-71), a member of the *Picornaviridae* family, has emerged as a critical non-polio neurotropic enterovirus. EV71 provokes acute neurological disease in children, which may result in cardiopulmonary failure and death. However, this non-enveloped, positive (+) strand RNA virus is a major cause of hand, foot, and mouth disease [31]. During the largest epidemic in Taiwan in 1998, more than 100,000 children were infected with EV-71 [32]. Despite extensive research from compound library screening to target-based chemical design, pharmaceutical developments have failed to meet pharmacological expectations [33].

The antiviral activity of three compounds, stachybogrisephenone B (**1**), grisephenone A (**2**), and 3,6,8-Trihydroxy-1-methylxanthone (**3**), which are new sesquiterpenoid and xanthone derivatives isolated from the cultures of sponge-derived fungus *Stachybotry* sp. HH1 ZDDS1F1-2, have been evaluated against *in vitro* replication of EV-71. The so-called compounds showed inhibitory activities against *in vitro* replication of EV71 with IC_50_ values of 30.1, 50.0 and 40.3 μM [34]. The efficiency of these cytotoxic compounds identified them as potential candidates for further studies towards drug discovery for EV-71 and other related viruses such as Coxsackie virus [35].

**Table 1:** Antiviral activities of marine fungi and their mechanism of action.

Virus	Antiviral Agent	Source		Chemical Class	Effect/Mechanism	Reference
EV71	Stachybogrisephenone B (**1**)	*Stachybotrys* sp.		Xanthone	IC_50_: 30.1 µM	[34]
	Grisephenone A (**2**)	*Stachybotrys* sp		Xanthone	IC_50_: 50.0 µM	[34]
	3,6,8-Trihydroxy-1-methylxanthone (**3**)	*Stachybotrys* sp		Xanthone	IC_50_: 40.3 µM	[34]
HSV	Halovirs A–E (**4**–**8**)	*Scytalidium* sp.		Peptide	direct inactivation	[36]
	11a-dehydroxyisoterreulactone A (**9**)	*Aspergillus terreus* SCSGAF0162		Lactone	IC_50_: 33.38 μM	[37]
	Arisugacin A (**10**)	*Aspergillus terreus* SCSGAF0162		Lactone	IC_50_: 12.76 μM	[37]
	Isobutyrolactone II (**11**)	*Aspergillus terreus* SCSGAF0162		Lactone	IC_50_: 62.08 μM	[37]
	Aspernolide A (**12**)	*Aspergillus terreus* SCSGAF0162		Lactone	IC_50_: 68.16 μM	[37]
	Balticolid (**13**)	*Ascomycetous* strain 222		Macrolide	IC_50_: 0.45 μM	[38]
HIV	Equisetin (**14**)	*Fusarium heterosporum*		Tetramic acid	IC_50_: 15 µM	[39]
	Phomasetin (**15**)	*Phoma* sp.		Tetramic acid	IC_50_: 10 µM	[39]
	Integric acid (**16**)	*Xylaria* sp.		Acylated eremophilane sesquiterpenoid	IC_50_: 10 µM	[40]
IFV	Stachyflin (**17**)	*Stachybotrys* sp. RF-7260	Sesquiterpenoidal alkaloid		inhibition of fusion between the viral envelope and the endosome	[41,42]
	Oxoglyantrypine (**18**)	*Cladosporium* sp.	Indole alkaloid		IC_50_: 85 µM	[43]
	Norquinadoline A (**19**)	*Cladosporium* sp.	Indole alkaloid		IC_50_: 82 µM	[43]
	Deoxynortryptoquivaline (**20**)	*Cladosporium* sp.	Alkaloid		IC_50_: 87 µM	[43]
	Deoxytryptoquivaline (**21**)	*Cladosporium* sp.	Alkaloid		IC_50_: 85 µM	[43]
	Tryptoquivaline (**22**)	*Cladosporium* sp.	Alkaloid		IC_50_: 89 µM	[43]
	Quinadoline B (**23**)	*Cladosporium* sp.	Alkaloid		IC_50_: 82 µM	[43]
	Cladosin C (**24**)	*Cladosporium sphaerospermum* 2005-01-E3	Hybrid polyketide		IC_50_: 276 μM	[44]
	(*Z*)-5-(Hydroxymenthyl)-2-(6′)-methylhept-2′-en-2′-yl)-phenol (**25**)	*A. sydowii* ZSDS1-F6	Bisabolane-type sesquiterpenoid		IC_50_: 57.4 µM	[45]
	Diorcinol (**26**)	*A. sydowii* ZSDS1-F6	Prenylated diphenyl ether		IC_50_: 66.5 µM	[45]
	Cordyol C (**27**)	*A. sydowii* ZSDS1-F6	Diphenyl ether		IC_50_: 78.5 µM	[45]
	Rubrolide S (**28**)	*A. terreus* OUCMDZ-1925	Rubrolide		IC_50_: 87.1 µM	[46]
	Asperterrestide A (**29**)	*A.terreus* SCSGAF0162	Cyclic tetrapeptide		H1N1 IC_50_: 15 µM; H3N2 IC_50_: 8.1 µM	[47]
	Isoaspulvinone E (**30**)	*A.terreus* Gwq-48	Butenolide		IC_50_: 101.23 µM	[48]
	Aspulvinone E (**31**)	*A.terreus* Gwq-48	Butenolide		IC_50_: 192.05 µM	[48]
	Pulvic acid (**32**)	*A.terreus* Gwq-48	Butenolide		IC_50_: 94.42 µM	[48]
	Emerimidine A (**33**)	*Emericella* sp. (HK-ZJ)	Isoindolone		IC_50_: 201.1 µM	[49]
	Emerimidine B (**34**)	*Emericella* sp. (HK-ZJ)	Isoindolone		IC_50_: 296.62 µM	[49]
	Purpurquinone B (**35**)	*P. purpurogenum* JS03-21	Azaphilone		IC_50_: 61.3 μM	[50]
	Purpurquinone C (**36**)	*P. purpurogenum* JS03-21	Azaphilone		IC_50_: 64.0 μM	[50]
	Purpuresters A (**37**)	*P. purpurogenum* JS03-21	Benzofuran		IC_50_: 85.3 μM	[50]
	TAN-931 (**38**)	*P. purpurogenum* JS03-21	Nonsteroidal aromatase		IC_50_: 58.6 μM	[50]
	Sorbicatechol A (**39**)	*P. chrysogenum* PJX-17	Sorbicillinoids		IC_50_: 85 μM	[51]
	Sorbicatechol B (**40**)	*P. chrysogenum* PJX-17	Sorbicillinoids		IC_50_: 113 µM	[51]
PRRS	Tetrahydroaltersolanol C (**41**)	*Alternaria* sp. ZJ-2008003	Anthraquinone derivatives		IC_50_: 65 µM	[52]
	Alterporriol Q (**42**)	*Alternaria* sp. ZJ-2008003	Isoindolone derivatives		IC_50_: 39 µM	[52]
MCV	Sansalvamide A (**43**)	*Fusarium* sp.	Pentadepsipeptide		inhibition of topoisomerase	[53]
RSV	22- *O*-(*N*-Me-l-valyl)-21-epi-aflaquinolone B (**44**)	*Aspergillus* sp. XS-20090B15	Prenylated dihydroquinolone derivatives		IC_50_: 42 nM	[54]
TMV	2-(4-hydroxybenzyl) quinazolin-4(3*H*)-one (**45**)	*P. oxalicum* 0312F_1_	Alkaloid		EC_50_: 399.57 µM	[55,56]
	2-(4-hydroxybenzoyl) quinazolin-4(3H)-one (**46**)	*P. oxalicum* 0312F_1_	Alkaloid		EC_50_ not tested	[55,56]
	Methyl 4-hydroxyphenylacetate (**47**)	*P. oxalicum* 0312F_1_	Ester		EC_50_: 829.15 µM	[55,56]
	AGI-B4 (**48**)	*Neosartorya fischeri* 1008F1	Dihydroxanthenone		IC_50_: 260 µM	[57]
	3,4-dihydroxybenzoic acid (**49**)	*Neosartorya fischeri* 1008F1	Polyphenol		IC_50_: 630 µM	[57]

### 2.2. Herpes Simplex Viruses (HSVs)

HSV-1 and HSV-2 are important and prevalent human pathogens from *Herpesviridae* family. These HSVs are characterized by four main constituents, namely an icosapentahedral capsid, an amorphous layer of proteins surrounding the capsid, an envelope, and an electron-dense core with large double-stranded DNA. HSV-1 is the main causative agent for orofacial infections such as cold sores, while HSV-2 is associated with a sexual transmitted infection (STI)—namely, genital herpes—that also can be transmitted to neonates during delivery through the infected mother’s birth canal. Despite numerous reports of natural products with inhibitory effects against HSV, commercial drugs are generally limited to synthetic antiviral agents with a natural product pharmacophore such as acyclovir, penciclovir, and famciclovir [58,59].

Halovirs A–E (**4**–**8**), a series of lipophilic linear peptides that were isolated from the genus *Scytalidium* during saline fermentation [40], exhibited *in vitro* antiviral activity against HSV-1 and HSV-2 in a time- and dose-dependent manner. This activity was suggested to be through viral membrane destabilization. These fungal metabolites exert direct virucidal activity against extracellular HSV particles as an applicable approach to the inhibition of HSV transmission [36].

Nong and colleagues (2014) [37] reported the antiviral activity of four compounds including 11a-dehydroxyisoterreulactone A (**9**), arisugacin A (**10**), isobutyrolactone II (**11**), and aspernolide A (**12**), with IC_50_ values against HSV-1of 33.38, 12.76, 62.08, and 68.16 μM, respectively. These compounds derived from a marine fungus, *Aspergillus terreus* SCSGAF0162, which was isolated from the South China Sea (18°11′ N, 109°25′ E) gorgonian corals *Echinogorgia aurantiaca*, under solid-state fermentation of rice.

A new 12-membered macrolide, balticolid (**13**), is one of the antiviral naphthalenone byproducts from the ethyl acetate (EtOAc) extract of the culture broth of fungal strain 222 belonging to the Ascomycota, which was obtained on driftwood gathered from the Greifswalder Bodden shore, Baltic Sea, Germany. To evaluate the antiviral activity of balticolid against influenza A virus and HSV-I, the non-cytotoxic concentrations of the compound were examined on *in vitro* replication of both viruses. Balticolid showed potent inhibitory effects against HSV-I with IC_50_ = 0.45 μM. However, there was no significant antiviral activity against *in vitro* replication of the influenza A virus [38].

### 2.3. Human Immunodeficiency Virus (HIV)

HIV causing acquired immunodeficiency syndrome (AIDS) is the major contributing factor for the elevation of infectious disease mortality all around the world [60]. This member of the *Retroviridae* family has a unique structure, which contains two copies of positive single-stranded RNA and a conical capsid. HIV has the potential to infect pivotal cells of the human immune system such as dendritic cells, macrophages, and mainly T helper cells. Until 2014, 28 antiretroviral drugs were on the market to treat HIV infection and classified based on their drug class into six main groups, integrase inhibitors, protease inhibitors, non-nucleoside reverse transcriptase inhibitors, nucleoside/nucleotide reverse transcriptase inhibitors, and entry inhibitors (fusion inhibitors and CCR5 agents) [61]. However, the annual expense of pharmaceutics for each individual can reach up to $24,000 in the US, depending on the group of anti-HIV drugs [62,63].

Equisetin (**14**) and its new enantiomeric homologue, phomasetin (**15**), isolated from the marine fungi *Fusarium heterosporum* and *Phoma* sp., respectively, exhibited *in vitro* inhibitory activity against the integrase enzyme of HIV-1, which is an important enzyme for the HIV-1 replication cycle. Further characterization of the active compound from a marine fungal species, *Xylaria* sp., yielded integric acid (**16**), which structurally is similar to equisetin (**14)** as a novel and unique class of integrase inhibitors [39]. These compounds prevent the amalgamation reactions catalyzed by pre-integration compounds derived from HIV-1-infected cells [24,64]. Integric acid ****impeded 3́end activity and strand transferal reactions, both with IC_50_ = 10 μM.

### 2.4. Influenza Virus

Every few years, emergence of a novel influenza virus with fatal pathogenicity attracts global attention to the marked numbers of afflicted patients and the high rate of death; still human beings are confounded by virus recombination [65]. Reformulation of the annual vaccine based on the expected upcoming influenza strains is always accompanied by some failures due to unforeseen pandemic viruses and antigenic mismatch [66]. Influenza A virus, a member of the *Orthomyxoviridae* family, is a negative (−) strand RNA virus and has several subtypes named based on the H (hemagglutinin) and N (neuraminidase) antigens. Birds are the main host for influenza A virus; however, transmission to domestic poultry may infect some mammals and provoke dangerous human influenza pandemics [67]. Respiratory droplets, aerosols, and direct contact with secretions are mentioned as the three main modes for viral transmission [68].

Stachyflin (**17**) was isolated from *Stachybotrys* sp. RF-7260 by the solid-state fermentation method. This terpenoid elicited an IC_50_ = 0.003 μM against influenza A virus (H1N1), which was comparable with other antiviral agents such as zanamivir and amantadine [41]. It is suggested that stachyflin, with a unique mechanism of action, suppresses the first stage of H1N1 and H2N2 viral infections (entering into the host cell through fusion between the viral envelope and endosomal membrane) [42]. This antiviral activity was confirmed in an *in vivo* study by oral administration of stachyflin and its derivatives with a solution in PEG [42].

In light of investigations for discovering anti-influenza compounds, an effective mangrove-derived fungal strain, *Cladosporium* sp., has been identified with potential for further study. A chemical analysis of the EtOAc decoctions of both the fermentation broth and mycelia of the fungus, which led to the isolation of six novel byproducts of glyantrypine and pyrazinoquinazoline (indole alkaloids), with potential antiviral property against H1N1 strain of influenza virus. The resultant compounds, oxoglyantrypine (**18**), norquinadoline A (**19**), deoxynortryptoquivaline (**20**), deoxytryptoquivaline (**21**), tryptoquivaline (**22**), and quinadoline B (**23**) revealed noteworthy antiviral activities against H1N1 strain with IC_50_ values of 85, 82, 87, 85, 89, and 82 μM, respectively [43]. In a later assay, Wu and colleagues (2014) [44] evaluated the secondary bioactive metabolites from a deep-sea-derived fungus, *Cladosporium sphaerospermum* 2005-01-E3, obtained from residues in the Pacific Ocean. The novel compound cladosin C (**24**) showed moderate antiviral activity against influenza A (H1N1) virus with an IC_50_ = 276 μM.

In addition, a recent study identified new bioactive natural compounds from a sponge-associated fungi, the ZSDS1-F6 strain of *Aspergillus sydowii*, which was isolated from an unknown marine sponge gathered from the Xisha Islands of China. The derived compounds, (*Z*)-5-(Hydroxymenthyl)-2-(6′)-methylhept-2′-en-2′-yl)-phenol (**25**), diorcinol (**26**) and cordyol C (**27**), exhibited minor antiviral activity against the influenza (H3N2) virus with IC_50_ values of 57.4, 66.5, and 78.5 μM, respectively [45]. Furthermore, two novel rubrolide compounds were isolated from the fermentation stock of the marine-derived fungus *Aspergillus terreus* (OUCMDZ-1925 strain) by Zhu and colleagues [46] ,of which rubrolide S (**28**) presented prominent antiviral activity against influenza A (H1N1) virus with an IC_50_ = 87.1 μM.

Asperterrestide A (**29**), a cyclic tetrapeptide compound with antiviral properties that derived from the fungal strain of *A. terreus* SCSGAF0162, was isolated from the tissue of the gorgonian *Echinogorgia aurantiaca*, collected from Sanya, Hainan Province, China. **29** displayed suppressive impacts on the influenza virus strains A/WSN/33 (H1N1) and A/Hong Kong/8/68 (H3N2) with IC_50_ values of 15 and 8.1 μM, respectively [47]. Further research was conducted on a new strain, *A.* *terreus* Gwq-48, which was derived from a mangrove rhizosphere soil sample in the coast of Fujian state. The analysis of its chemical ingredients resulted in the segregation of one novel aspulvinone, isoaspulvinone E (**30**), aspulvinone E (**31**), and pulvic acid (**32**), which exhibited notable anti-influenza A (H1N1) virus activities, with IC_50_ values of 101.23, 192.05, and 94.42 μM, respectively [48].

An investigation with the goal of producing an anti-influenza virus resulted in the isolation of a fungus verified as *Emericella* sp. (HK-ZJ), isolated from the internal rind of a mangrove plant *Aegiceras corniculatum* in the district of HaiKou, China [49]. Following the evaluation of ten compounds, emerimidine A and B (**33** & **34**), emeriphenolicins A and D, aspernidine A and B, austin, austinol, dehydroaustin, and acetoxydehydroaustin, for their *in vitro* effect against replication of H1N1 in MDCK cells, just two compounds, **33** and **34**, displayed average suppressive effects with IC_50_ values of 201.1 and 296.62 μM, respectively.

A recent study on novel drugs against the influenza virus from extremophiles, an acid-tolerant fungal strain JS03-21, determined as *Penicillium purpurogenum*, was derived from the regional red soil by the Lujiang River from Jianshui, Yunnan, China. Subsequently, the antiviral activity was detected for compounds purpurquinones B and C (**35** & **36**), purpuresters A (**37**), and TAN-931 (**38**) against H1N1, with IC_50_ values of 61.3, 64.0, 85.3, and 58.6 μM, respectively [50]. In a further late study conducted by Peng and colleagues [51], on the same genus but a different strain called *Penicillium* *chrysogenum* PJX-17, two novel compounds, sorbicatechols A and B (**39** & **40**), were properly isolated from a sorbicillin component and a styrene moiety by endo- and exo-Diels—Alder cycloadditions, and both of them showed anti-H1N1 activity, with IC_50_ values of 85 and 113 μM, respectively.

### 2.5. Porcine Reproductive and Respiratory Syndrome Virus (PRRSV)

PRRSV is a member of the *Arteriviridae* family with a complex epidemiological profile due to the persistence of the virus in tissue even several months after the acute stage. This small, enveloped, positive (+) strand RNA virus infects pigs and causes respiratory illness and a major failure in the reproduction of sows. An RNA genome of about 15 kilobases in size is responsible for the coding of two principal polyproteins, namely 1a and 1ab [69]. PRRSV classifies into two subgroups, namely A and B, representing the North American and European strains, which have different level of virulence. Similar to the other viruses in this family, the PRRS virus targets macrophages [54]. Assorted methods of PRRSV transmission via different fomites such as equipment, hands, needles, and footwear associated with mechanical transmitters, including flies and mosquitoes, increase the high risk of PRRSV infection [70]. Despite the prevalence of vaccines, control of the virus infection in usual swine conditions has always been a major hindrance to food industries [54].

A recent investigation by Zheng and colleagues [52] reported two marine-derived compounds with antiviral potential against PRRSV. Tetrahydroaltersolanol C (**41**) and alterporriol Q (**42**) were isolated from the marine-derived fungus ZJ-2008003, obtained from Sarcophyton sp., a soft coral in the South China Sea. Molecular and morphological characterizations identified the isolated species as an Alternaria sp. The compounds **41** and **42** revealed antiviral activities against PRRSV with IC_50_ values of 65 and 39 μM, respectively [52].

### 2.6. Molluscum Contagiosum Virus (MCV)

As a member of the *Poxviridae*, MCV has a unique mode of replication in the human epidermis: itprecipitates a common skin infection, especially in children, through augmentation of epidermal cell mitosis and disruption of cell differentiation. However, similar to other species in this family, this linear, double-stranded DNA virus is characterized by lateral bodies, a core, an envelope, and a surface membrane. The viral genome and replication afflicts several cellular pathways, including cell death, cell cycle, inflammation, and innate immunity. To escape from immune surveillance, the MCV virus does not pass the basement membrane of the epidermis. MCV skin diseases can be comfortably recognized, and a combination of topical treatment and local physical therapy can efficaciously obliterate the virus infection [71].

Sansalvamide A (**43**), isolated from a marine fungus, *Fusarium* sp. [72] as a cyclic depsipeptide, elicited inhibitory activity against topoisomerase of MCV. The antiviral effect against this pathogen (IC_50_ = 124 μM) was induced through suppression of topoisomerase-mediated DNA-binding, DNA relaxation, and formation of covalent complex [53]. Due to close correlation between MCV lesions and AIDS patients, this antiviral activity was considered a noteworthy bioactivity for marine fungi [73]. Hence, development of cyclic depsipeptide derivatives from sansalvamide A may catalyze the discovery of new generations of anti-MCV agents.

### 2.7. Respiratory Syncytial Virus (RSV)

As a member of the *Paramyxoviridae* family, RSV is responsible for the most abundant infection in the lower respiratory tract of young children and infants and causes approximately 100,000 pediatric infections with 250 deaths each year in the United States [74]. This negative (−) strand RNA virus with approximately 15 kilobases has 10 genes, which are responsible for the production of 11 viral proteins. The infection process is triggered through binding to host cell receptors by G protein. Following the attachment as a surface viral protein, F protein causes the combination of nearby cells and subsequent formation of “syncytia,” which inspired the name of the virus [75]. There are only limited therapeutic agents such as palivizumab and ribavirin that are reported against RSV, and this fact illustrates the urgent need for the development of new anti-RSV drugs [74].

Screening of an extract isolated from *Aspergillus* sp. XS-20090B15, which has been derived from the gorgonian *Muricella abnormaliz* from the South China Sea and cultured on a rice medium, revealed anti-RSV activity. Compound 22-*O*-(*N*-Me-l-valyl)-21-epi-aflaquinolone B (**44**) exhibited exceptional anti-RSV activity with an IC_50_ value of 42 nM, roughly 500 times more potent than ribavirin, the positive control used in this study, with IC_50_ = 20 μM [54]. In addition, aflaquinolone D, another compound of the same fungus, showed anti-RSV activity but not as potent as the former (IC_50_ = 6.6 μM).

### 2.8. Tobacco Mosaic Virus (TMV)

As a plant virus, TMV markedly debilitates the horticulture and agriculture industries [76]. This rod-like virus has a complex capsid and positive (+) strand RNA with approximately 6.4 kilobases. TMV infection creates particular distinguishing features on plants, including leaf discoloration and mosaic mottling [77]. Infection of more than 400 assorted plant species from 36 families, including cucumber, potato, tomato, and tobacco, foregrounds the importance of TMV. However, after infection by a virus, there is no definite treatment for the TMV infections [78,79,80]. Due to the possible side effects of synthetic drugs on plants, natural product derivatives can be more compatible with infected hosts.

Recent surveys reported that the extract of strain 0312F_1_ from *Penicillium oxalicum* exhibited a strong inhibitory activity against the replication of TMV [55,56]. Bioactivity analyses indicated that 2-(4-hydroxybenzyl) quinazolin-4(3*H*)-one (**45**) and methyl 4-hydroxyphenylacetate (**47**) possessed strong suppressive activity against TMV with EC_50_ values of 399.57 μM and 829.15 μM, respectively; while, compared to those, the new compound 2-(4-hydroxybenzoyl) quinazolin-4(3*H*)-one (**46**) revealed average suppressive activity against TMV (EC_50_ was not tested) [49]. Prior to this study, Tan and colleagues (2012) [57] reported the antiphytoviral properties of marine-derived fungus *Neosartorya fischeri* strain 1008F1. The bioactive assays signifying those two compounds, AGI-B4 (**48**) and 3,4-dihydroxybenzoic acid (**49**), showed strong inhibitory impact on the replication of tobacco mosaic virus (TMV), with IC_50_ values of 260 and 630 μM, respectively.

## 3. Conclusions

The increasing rate of viral resistance to antiviral drugs and drug toxicity is becoming a challenging problem in antiviral therapy. There are numerous reports on the resistance of different viruses to approved antiviral drugs [81,82,83]. However, there are many viral infections without any available effective treatment. Therefore, natural products from different living organisms including marine organisms could be potential candidates for development of new antiviral drugs. As we summarized in this review, there are different biomolecules from different chemical categories containing peptides, alkaloids, terpenoids, diacyglycerols, steroids, polysaccharides, and even more from different marine fungi with significant antiviral activities, as shown especially through *in vitro* studies [47,84,85]. Therefore, further investigation towards *in vivo* and even pharmacological studies for some of the abovementioned effective compounds seems to be crucial.

Organisms inhabiting the marine environment provide a diversity of bioactive compounds, which are exclusive as the aqueous habitat demands molecules with particular and vigorous biological compounds. Many scholars are devoted to investigating marine organisms to determine the development of potential biomolecules into therapeutic drugs and numerous compounds from marine fungi have been shown to possess notable antiviral activities. On the other hand, there are many important animal and human viruses yet to be studied, since for most viral diseases there has not been any effective therapeutic treatment available thus far. Therefore, infectious viruses with widespread prevalence, including EV71, HSV, HIV, MCV, and RSV, were used to examine the antiviral potential of the isolated compounds. Moreover, viruses responsible for important plant and animal infections, namely TMV and PRRS, were also employed in several studies. All together, the results showed quite noticeable cytotoxic effects against the respective viruses. From the preceding statement, we presented various compounds isolated from different marine fungi genera of which the most important ones exploited for their antiviral potential were *Aspergillus* sp., *Penicillium* sp., *Cladosporium* sp., *Stachybotrys* sp., and *Neosartorya* sp.; they are summarized in Table 1. Among these compounds, **13**, a newly derived strain of Ascomycete, revealed marked inhibitory activity against HSV. Furthermore, **17** prompted a potent anti-influenza virus activity by showing a very low IC_50_ value (0.003 µM) and also oral administration of **17** in an *in vivo* study in PEG confirmed its significant antiviral activity. Furthermore, **44** exhibited a notable potency to elicit substantial antiviral activity against RSV. Nonetheless, the majority of the investigations were limited to basic screening and no mechanism of action was established for active compounds. It is pivotal for further research to characterize and determine the virus or host factors, which were targeted by antiviral compounds.

To develop antiviral drugs derived from marine fungi, *in vivo* and clinical studies are other aspects that should be exploited. The variety of the natural products from marine fungi evidently determines the potential for assigning some selected compounds to *in vivo* and probably clinical trials for forthcoming progress of anti-infective drugs. One of the considerable upcoming challenges will be the extensive production of these compounds to meet the demand for clinical trials and drug development. Several investigators believe that a specific form of combined genetic and metabolic engineering will be the potential resolution for commercial manufacture of these compounds [3]. It is hoped that this review could be a helpful source of guidance towards the discovery of new antiviral drugs.
